# Intramammary Infusion of Micronised Purified Flavonoid Fraction (MPFF) in Mastitis-Diagnosed Dairy Cows Naturally Infected by *Staphylococcus* spp. in the Late Lactation

**DOI:** 10.3390/vetsci10050335

**Published:** 2023-05-06

**Authors:** Miguel A. Gutiérrez-Reinoso, José B. Uquilla, Jorge L. Guamaní, Ángel E. Caiza, Rocío P. Carrera, Manuel Garcia-Herreros

**Affiliations:** 1 Carrera de Medicina Veterinaria, Facultad de Ciencias Agropecuarias y Recursos Naturales, Universidad Técnica de Cotopaxi (UTC), 0501491 Latacunga, Ecuador; jorge.guamani3@utc.edu.ec (J.L.G.); angel.caizamvz@gmail.com (Á.E.C.); rocio.carrera1702@gmail.com (R.P.C.); 2 Departamento de Ciencia Animal, Laboratorio de Biotecnología Animal, Facultad de Ciencias Veterinarias, Universidad de Concepción, Chillán (UdeC) 3780000, Chile; 3 Departamento de Asesoría Ganadera, La Holandesa SAS, Quito 170179, Ecuador; mvzjoseu@outlook.es; 4 National Institute for Agricultural and Veterinary Research (INIAV, I.P.), Polo de Santarém, Quinta da Fonte Boa, 2005-048 Santarém, Portugal

**Keywords:** MPFF, flavonoids, intramammary infusion, mastitis, late lactation, dairy cows

## Abstract

**Simple Summary:**

Infectious mastitis causes significant economic losses and affects dairy cattle health and welfare. The present research aimed to test an intramammary-infused therapeutic compound to promote sustained mastitis treatment and improve cure rates in dairy cattle. The effects of intramammary infusion of micronised purified flavonoid fraction (MPFF) were studied as a natural alternative to conventional synthetic antimicrobials and against antimicrobial resistance in mastitis-diagnosed dairy cows infected by *Staphylococcus* spp. in the late lactation.

**Abstract:**

Infectious mastitis is the most prevalent health problem in dairy cattle that can result in permanent economic losses on dairy farms. The micronised purified flavonoid fraction (MPFF) is a biocompatible active polyphenolic compound derived from flavonoid glycosides which exhibits several antimicrobial, anti-inflammatory, and phlebotonic properties. The goal was to assess the effects of an alternative therapy for mastitis based on MPFF intramammary infusions in late lactation in dairy cows naturally infected by *Staphylococcus* spp. The California Mastitis Test (CMT scores) was performed to detect mastitis-positive quarters in twelve dairy farms. All cows were screened for immune response by measuring somatic cell counts (SCCs; cells/mL) in milk samples from each quarter. In addition, bacteriological identification, pathogenic bacterial isolates, and total bacterial counts (TBCs; CFU/mL) were assessed before (day 0, last milking day) and after (day 3 post-calving) MPFF application. Antimicrobial sensitivity patterns of the pathogenic isolated bacteria were evaluated. Finally, cure rates (%) were determined for each MPFF treatment. Around 15 mastitis-related genera were isolated. *Staphylococcus aureus* (25.2%) and coagulase-negative Staphylococci (CNS; 22.4%) were the most prevalent pathogens. No statistical differences were observed in SCCs and TBCs after low, medium, and high MPFF dose administration in *S. aureus*-positive mastitis cases (*p* > 0.05). However, differences were observed in SCCs and TBCs after medium and high MPFF dose administration in CNS-positive quarters (*p* < 0.05). The pathogenic bacteria isolate reduction after MPFF applications showed a dose-response fashion (*p* < 0.01) while isolates obtained from controls and low MPFF-treated quarters remained similar, irrespective of the pathogen (*p* > 0.05). Sensitivity patterns were variable, although *S. aureus* remained resistant, irrespective of the MPFF dose. However, CNS showed a dose-response sensitivity pattern. Finally, the cure rate (%) on day 3 post-partum improved significantly using medium and higher MPFF doses in CNS-positive quarters (*p* < 0.05). In conclusion, MPFF treatment was found to be more effective for CNS-positive cases in the late lactation due to noticeable dose-specific responses regarding somatic cells, bacterial counts, sensitivity patterns, and cure rates in dairy cattle.

## 1. Introduction

Mastitis is the most widely diagnosed and prevalent health issue in the dairy industry and it has the largest economic impact on dairy farms [1,2,3]. It consists in an inflammatory process of the mammary gland most often caused by infectious agents [4]. Most mastitis is categorised as mild (only abnormal milk) [5]. Moreover, mastitis incurs significant costs related to diagnosis, treatment, production losses, animal culling, long withdrawal periods, and discarded milk due to antimicrobial residues [6]. The mastitis prevention programs are costly and the intramammary infections affect animal welfare [7,8]. The intramammary infusion of antimicrobials is part of the mastitis prophylaxis programs in dairy cattle; however, the antimicrobial over-use increases the risk of selection for resistant pathogens in livestock [9]. Although antimicrobial agents aim to cure existing infections and prevent new infections during the dry period, they are not equally effective against all pathogens [10]. The resistance against β-lactam antimicrobials may occur as a consequence of mutations of the bacterial cell wall [11].

Based on the bacteriological etiologic agent, mastitis can be classified into a contagious and environmental disease [4,12,13]. *Staphylococcus* spp. is the most common cause of mastitis, causing several signs of illness [14,15]. A high percentage of *Staphylococcus* spp. isolates from bovine mastitis cases were described to be antimicrobial-resistant, depending on the pathogen species and the antibiotic used [16]. *Staphylococcus aureus* has been considered as among the most frequently isolated contagious pathogen in mastitis cases with poor cure rates and recurrent infections [17]. *S. aureus* is known to be highly resistant to antibiotics because the pathogen forms biofilm in the mammary gland tissues, survives in the host cells and it has the ability to sequester itself within micro-abscesses, evading the immune system [18,19,20]. Recently, coagulase-negative Staphylococci (CNS) have become the most common environmental bovine mastitis pathogens [21,22]. CNS are capable of interacting with fibronectin-binding protein and produce mucoid extracellular substances and toxins [23,24]. Some of CNS-derived infections may persist for long periods of time [25,26].

The California Mastitis Test (CMT) provides an immediate result and is and inexpensive method [27]. A positive CMT is considered indicative of an increase in somatic cells and suggests the presence of intramammary infection [28,29] using a scale of 0, trace, 1, 2, or 3 depending on the mastitis severity [30,31]. Similarly, the somatic cell count (SCC) has traditionally been used to define intramammary infection [28]. SCC consist of polymorphonuclear neutrophil leukocytes (PMN > 95%), macrophages, lymphocytes, and epithelial cells [32,33,34]. An increase of phagocytes in the infected mammary gland contributes to high SCC [35]. Moreover, total bacterial counts (TBCs) and in vitro tests are frequently used to test the effectivity of different antibiotic substances [36].

A high percentage of environmental mastitis diagnosed cases within 100 d of calving have been attributed to late lactation infection [37]. The treatment of subclinical mastitis cases in the late lactation by intramammary infusion of antimicrobials is a frequent strategy in the dairy industry because bovine mastitis prevalence has soared in recent years; however, the bacteriological cure rate is variable [38,39,40]. During the late lactation, the level of antibacterial components and concentration of immune cells in the milk are minimal [41]. Identification and treatment of infected cows late in lactation could help to improve mastitis control in the next lactation [42,43,44]. Moreover, there are some advantages, such as a decrease in the amount of discarded milk, low risk of antibiotic residues, no withdrawal period, and improved milk quality and quantity in the next lactation [43,45].

The micronised purified flavonoid fraction (MPFF; diameter <2 µm) is a natural biocompatible active polyphenolic compound derived from flavonoid glycosides which exhibits several biological properties such as antimicrobial, anti-inflammatory, and phlebotonic [46,47,48]. It consists of 90% micronised diosmin (diosmetin 7-rhamnoglucoside; C_28_H_32_O_15_; MW: 608.6 Da) and 10% expressed as micronised hesperidin (hesperitin 7-rhamnoglucoside; C_28_H_34_O_15_; MW: 610.6 Da). MPFF has been applied for different therapies in humans and in other mammals [49,50]. MPFF is a well-known radical scavenger with beneficial effects modulating key enzymes [50]. MPFF is considered to have no harmful effects, such as residues in milk, unlike other antimicrobial agents, such as oxytetracycline, among others [50,51].

Thus, the objective of the present research was to study the potential therapeutic effects of the intramammary infusion of micronised purified flavonoid fraction (MPFF) in mastitis-diagnosed dairy cows naturally infected by *Staphylococcus* spp. in late lactation. Therefore, the present study aimed to develop a therapeutic MPFF-formulation that could be intramammary infused as an alternative to conventional synthetic antimicrobials with the aim of promoting sustained mastitis treatment and improving cure rates. For this purpose, pathogen identification, potential changes in SCCs response, total bacteria counts (TBCs), antibiotic sensitivity, and the bacteriological cure will be carried out as indicators of MPFF therapeutic-protective derived effects.

## 2. Materials and Methods

### 2.1. Media and Reagents

All media and reagents were purchased from Sigma (Sigma-Aldrich, St. Louis, MO, USA) unless otherwise stated.

### 2.2. Animals and Dairy Management

The research was performed on adult lactating Holstein-Friesian breed cows (2nd–5th lactation) calved during a 3-year period (January 2017 to December 2019). The cows belonged to 12 adjacent commercial dairy herds consisting of 196 ± 32 cows per farm located in Canton Mejía, Province Pichincha, Ecuador. Cows (~2350; BCS ≥ 2.5) were rotationally grazed through the pasture (*Lolium perenne* and *Trifolium repens ad libitum* intake) following standard management practices and were fed an adjusted total mixed ration (TMR twice daily: corn silage, grass silage, and concentrate) with water *ad libitum*. All cows were routinely milked twice daily (at 6 a.m. and at 6 p.m.) and the annual average milk production was 3000–4000 kg per cow/year. All cows were checked weekly by veterinary routine services to maintain the herd-health control. Milk production parameters (volume, protein, fat, and somatic cell count) were recorded by the Central Laboratory for Milk Recording of the Ministry of Agriculture and Livestock (MAG-AGROCALIDAD, Tumbaco, Ecuador) after milk sampling once per week. The individuals enrolled in the study were late lactation cows (~7–8 mo. post-calving) and produced ~10–15 kg milk/day. The cows were dried off by the reduction of feed (TMR fed once daily) and intermittent milking for 12 days with the daily milk production at the last day of milking being around 4–6 kg milk/cow. In the present study, subclinical mastitis implies a CMT+ (traces/grade I), high SCC (>200,000 cells/mL) together with the identification of at least one pathogenic species. The mammary gland health status was monitored in the late lactation (4 weeks before drying-off) and the enrolled quarters were confirmed twice (two consecutive weeks) before treatment by CMT results, SCCs values, bacteriological tests, and TBCs values.

### 2.3. Milk Sample Collection

Milk samples from individual quarters were collected following a standard method applied for milk sampling near the end of lactation when the cows were on average 192 ± 21 days in milk (DIM). Cows were selected from each enrolled herd that had had at least two lactations and had not been treated with any antimicrobial before (data obtained from the electronic database). Duplicate milk samples (~5 mL) were collected from each quarter following a teat and teat orifice cleaning and disinfecting. All milk samples were collected immediately prior to infusion and on day 3 post-calving.

### 2.4. CMT Procedure

The glandular secretion was monitored once weekly before milking using the CMT [27,29,52]. The CMT results were scored on a scale of 0, trace, 1, 2, and 3 based on the appearance of the liquid fraction mixed, where 0 = no thickening, trace = slight thickening, 1 = thickening but not gel formation, 2 = slow thickening with gel formation, and 3 = rapid gel formation. All CMT scores were performed by the same technician.

### 2.5. SCCs Assessment

All cows were screened for infection by measuring SCCs in milk from each mammary gland. The mastitis-positive cows had individual SCCs of >200,000 cells/mL which was monitored two consecutive weeks by using an approved flow cytometry method (Foss Analytics, Hillerød, Denmark). The SCC cut-off was selected based on the National Mastitis Council (NMC, 2001) guidelines. Duplicate milk samples (~5 mL per sample) were collected from each quarter into clean sterile polyethylene labeled vials. The samples were held at room temperature for a maximum of 2 h before being stored at −20 °C until processing.

### 2.6. Milk Sample Culture and Bacteriological Analysis

A second milk sample for culture was taken immediately prior to the time of milking, for later evaluation of bacteriological cure. The milk samples were kept at 4 °C during transport to the laboratory and processed within 4 h in the Central Laboratory for Microbiology Diagnosis from the Ministry of Agriculture and Livestock (MAG-AGROCALIDAD, Tumbaco, Ecuador). Microbiological culture techniques were carried out immediately following the National Mastitis Council guidelines [53]. Briefly, 10 μL of milk was collected aseptically for culture (duplicate) and placed onto a 5% defibrinated blood agar plate containing 0.1% esculin [54,55] and incubated at 37 °C. Plates were checked for growth and hemolytic patterns after 24–36 h. Gram-negative rods were cultured onto MacConkey’s agar, and conventional biochemical tests were carried out for isolate identification, including indole, ornithine decarboxylase reactions, and through growth on triple sugar iron slants. Another differential identification was carried out using a coagulase test which was performed if only a β-hemolytic zone was detected around the colonies. A coagulase-positive result led to the classification of the pathogen as a *S. aureus;* however, if the coagulase test appeared as negative, the pathogen was classified as a CNS. A quarter was diagnosed as infected when at least one plate showed ≥1 CFU (*S. aureus*) or ≥2 CFU (CNS) of a single pathogen species. Plates with more than two species were considered contaminated. The bacteriologic cure of any existing infection was defined as the absence of isolation of the pathogen at the post-calving milk sampling (duplicate samples). Quarters which had the same microorganism isolated from the same quarter from both pre-treatment samples were included for bacterial cure evaluation. Finally, in vitro antimicrobial sensitivity tests were performed using culture antimicrobial discs on agar plates [diosmin-hesperidin (450 + 50 mg; 900 + 100 mg; 1350 + 150 mg)]. All isolates were categorised as either susceptible or resistant to every single antimicrobial. The growth inhibition zones were interpreted to record the level of sensitivity after 24–36 h at 37 °C in aerobic/anaerobic conditions.

### 2.7. Control Groups

A within-herd randomised controlled trial research was performed on the basis of mastitis detection. For the first control groups (negative controls; *n* = 25 quarters each group), only clinically healthy mastitis-free cows were enrolled (cows without history of mastitis), avoiding any pathology which could directly or indirectly affect the udder functionality. Only milk samples containing SCCs < 200,000 cells/mL and culture-negative cases defined as milk samples that did not result in the growth of any bacteria on blood or MacConkey agar were randomly assigned to negative control groups. For the second control groups (*S. aureus*- and CNS-positive controls; *n* = 25 quarters each), only cows displaying mastitis episodes (infection status was based on culture results from milk samples collected 4 days prior to drying-off) were considered (Table 1).

### 2.8. MPFF Intramammary Infusion Treatments

The quarters were divided into ten groups (controls and treatment groups). All treatments were initiated after the last milking and were pre-assigned using a random number generator. All experimental groups in the present study are shown in Table 1.

Quarters included in controls (negative and positive) received an intramammary infusion of 20 mL of sterilised saline solution instead (saline solution 0.9%). Intramammary infusion was applied twice after the last milking at 12-h intervals within the same day (24 h) using a disposable cannula and a plastic single-use 20 mL syringe. The low MPFF dose group received a single intramammary infusion of 20 mL of a water-based suspension of 500 mg (intramammary suspension of 450 mg diosmin + 50 mg hesperidin); The medium MPFF dose group was administered a single intramammary infusion of 20 mL of the same suspension but containing 1000 mg (900 mg diosmin + 100 mg hesperidin); and finally, the high MPFF dose group received 1500 mg (1350 mg diosmin + 150 mg hesperidin).

The active principle was dispersed up into the mammary gland via gentle massage of the teat and udder. Following infusion, all teat ends were sprayed with a 0.25% iodine-based antiseptic. All cows underwent a clinical examination throughout the experimental period by mammary gland palpation and were subjected to pre-treatment (pre-drying-off) and post-treatment (on day 3 post-calving) CMT, SCCs, and TBCs assessment.

### 2.9. Statistical Analyses

All data were obtained from different trial groups (MPFF-treated and non-MPFF-treated cows from both controls). Data were logarithmically transformed (including SCC to SCS) in order to remove heterogeneity of variance when an extreme heterogeneous variation among values was detected, as recommended by [56]. Due to no differences being observed regarding the level of significance when SCC and SCS were analysed and compared, the variable SCS was only used for the interpretation of the analysis while the variable SCC was used to show the analysed results. To analyse the differences from quarter-derived results before and after MPFF treatments, an ANOVA test (partitioned variance on a between- and within-animal group basis) was performed. Duncan and Bonferroni tests were carried out (corrections for between-animal group multiple comparisons) when significant interactions were observed. Fisher’s test was performed to assess the differences regarding clinical and bacteriological findings. Wilcoxon test/U Mann–Whitney tests were performed comparing pairs when an association between the nominal and ordinal variables was detected. A Chi-square test was carried out to test the differences in bacterial contamination among groups. The association between the different experimental groups was tested. Discrete independent variables were treatment group and cow. Each individual cow was used as the experimental unit. Data were grouped, taking into account the mastitis disease: healthy vs. mastitis-diagnosed cows. Intramammary infection and treatment status was included in the models (independent variable) and disease conditions were tested as possible confounders. Statistical analysis was performed using the SPSS v.25 software (SPSS Inc., Chicago, IL, USA). A *p* value of 0.05 or less was considered statistically significant.

## 3. Results

### 3.1. Identification of Mastitis Etiological Agents (Genera and Species) in Dairy Cows Naturally-Infected in the Late Lactation

Around 15 gram-positive and gram-negative genera, including Staphylococcus genus, were observed and were classified according to their potential pathogenicity (Table 2). Moreover, pathogen prevalence (total % and mastitis-positive %) was calculated from milk-derived samples. The most frequent isolates obtained during the pre-treatment screening samples (mastitis-diagnosed quarters 4 wk. before the beginning of the treatments) belonged to the following genera/spp.: *Staphylococcus aureus* (25.2%), *Escherichia coli* (20.4%), coagulase negative Staphylococci (CNS; 18.4%), Coliforms (15.6%), *Streptococcus agalactiae* (15.2%), *Streptococcus dysgalactiae* (12.8%), and *Staphylococcus chromogenes* (10.4%). Other isolated genera/species percentages were < 10% (Table 2). From the total species/types isolated, all were considered mastitis-related pathogenic species (15/15; 100.0%). However, 11 genera were considered non-frequent in developing mastitis cases (11/15; 73.3%).

### 3.2. Effects of MPFF Intramammary Infusion on SCCs Obtained in Dairy Cows Naturally-Infected by Staphylococcus spp. in the Late Lactation

After SCCs assessment by using milk samples, divergent profiles were observed depending on the sampling point and experimental group analysed. All milk samples were evaluated showing SCs increased counts, especially in mastitis-diagnosed quarters on day 0 (last milking day before drying-off) where the 100% of samples showed increased SCC >340 × 10^3^ cells/mL which is a frequent count in subclinical mastitis cases. Then, cellular evidence of subclinical inflammation/infection was observed in 100% of samples, irrespective of the time point or group assessed, with the negative controls being the lowest (~100 × 10^3^ cells/mL). The overall SCC (cells/mL) in *S. aureus*-positive cases at the first milk sample collection (day 0) was ~390 × 10^3^ cells/mL (range from 340–440 × 10^3^ cells/mL) versus ~400 × 10^3^ cells/mL (range from 320–500 × 10^3^ cells/mL) on day 3 after calving, considering just mastitis-diagnosed groups together (positive control and MPFF-treated cows). No differences were observed in SCCs after low, medium, and high MPFF dose administration in *S. aureus*-positive mastitis cases (*p* > 0.05). The overall SCC (cells/mL) in CNS-positive cases at the first milk sample collection (day 0) was ~400 × 10^3^ cells/mL (range from 380–460 × 10^3^ cells/mL) versus ~320 × 10^3^ cells/mL (range from 210–410 × 10^3^ cells/mL) on day 3 post-calving, considering only mastitis-diagnosed groups together (positive control and MPFF-treated cows). Statistical differences were observed in SCCs after medium and high MPFF dose administration in CNS-positive mastitis cases (*p* < 0.05). When SCCs were compared between *S. aureus*-positive vs. CNS-positive mastitis cases, statistical differences were observed in SCCs between both after medium and high MPFF dose administration (*p* < 0.05; Table 3).

### 3.3. Effects of MPFF Intramammary Infusion on the Number of Pathogen Isolates Obtained in Mastitis-Positive Quarters Naturally-Infected by Staphylococcus spp. in the Late Lactation

Differential pathogen isolate frequencies were obtained from positive MPFF-treated group samples in both *S. aureus*-positive vs. CNS-positive mastitis cases. Pathogenic bacteria isolates including Staphylococci (coagulase-positive *S. aureus* and coagulase-negative Staphylococci) were only isolated from the mastitis-diagnosed individuals (100%) when compared to negative control quarters having neither clinical signs nor pathogen isolates detected (0%). The isolate frequency did not differ in MPFF-treated compared to both positive control groups on day 0 (*p* > 0.05; Table 4). After MPFF application (day 3 post-calving), the frequencies differed when positive controls and MPFF-treated groups were compared, being lower in MPFF-treated quarters, irrespective of the pathogen (*p* < 0.05). Thus, the decreased frequencies from pathogens on day 3 post-calving (MPFF post-treatment) were related to a decreased score for CNS as well as with maintained frequency or non-growth changes for *S. aureus* (Table 4). The overall number of isolates obtained in *S. aureus*-positive cases at the first milk sample collection (day 0) was ~63 (range from 59–71 isolates) versus ~49 (range from 33–64 isolates) at the last collection (day 3 after calving) considering only mastitis-diagnosed groups together (positive control and MPFF-treated cows). Moreover, in *S. aureus*-positive cases, the occurrence of bacterial isolates was higher in positive control [60/25 (2.4)] compared to high dose MPFF-treated quarters [33/25 (1.3)] on day 4 post-partum (*p* < 0.05). In addition, differences were observed in the number of isolates obtained after medium and high MPFF dose administration in *S. aureus*-positive mastitis cases (*p* < 0.05). The overall number of isolates obtained in CNS-positive cases at the first milk sample collection (day 0) was ~61 (range from 57–66) versus ~33 (range from 17–45 isolates) at the last collection (day 3 after calving), considering only mastitis-diagnosed groups together (positive control and MPFF-treated cows). Statistical differences were observed in the number of isolates after low, medium, and high MPFF dose administration in CNS-positive mastitis cases (*p* < 0.05). Besides, in CNS-positive cases, the occurrence of bacterial isolates was significantly higher in the positive control (45/25 (1.8)) compared to high dose MPFF-treated quarters [17/25 (0.7)] on day 4 post-partum (*p* < 0.05). Finally, when the number of isolates were compared between *S. aureus*-positive vs. CNS-positive mastitis cases, statistical differences were observed in the number of isolates between both pathogens after low, medium, and high MPFF dose administration (*p* < 0.05; Table 4).

### 3.4. Effects of MPFF Intramammary Infusion on the Total Bacterial Counts (TBCs) Obtained in Mastitis-Positive Quarters Naturally-Infected by Staphylococcus spp. in the Late Lactation

Distinctive bacteriological profiles (TBCs) were obtained from positive MPFF-treated group samples in both *S. aureus*-positive vs. CNS-positive mastitis cases. The overall TBCs obtained in *S. aureus*-positive cases at the first milk sample collection (day 0) was ~3.61 × 10^5^ CFU/mL (range from 3.32–3.80 × 10^3^ CFU/mL) versus ~3.58 × 10^5^ CFU/mL (range from 3.16–4.22 × 10^5^ CFU/mL) at the last collection (day 3 after calving), considering only mastitis-diagnosed groups together (positive control and MPFF-treated quarters). No differences were observed in the TBCs obtained after low, medium, or high MPFF dose administration in *S. aureus*-positive mastitis cases (*p* > 0.05). The overall TBCs obtained in CNS-positive cases at the first milk sample collection (day 0) was ~3.73 × 10^5^ CFU/mL (range from 2.89–3.87 × 10^5^ CFU/mL) versus ~2.49 × 10^5^ CFU/mL (range from 1.72–3.12 × 10^5^ CFU/mL) at the last collection (day 3 post-calving), considering only mastitis-diagnosed groups together (positive control and MPFF-treated quarters). Statistical differences were observed in the TBCs after medium and high MPFF dose administration in CNS-positive mastitis cases (*p* < 0.05). When the TBCs were compared between *S. aureus*-positive vs. CNS-positive mastitis cases, statistical differences were observed in TBCs between both pathogens after low, medium, and high MPFF dose administration (*p* < 0.05; Table 5).

### 3.5. Antimicrobial Sensitivity Patterns of the Pathogenic Isolated Bacteria Obtained in In Vitro Cultures Derived from Milk Samples in the Late Lactation

Table 6 shows the contrasting antimicrobial sensitivity patterns of the isolated pathogens in in vitro cultures derived from milk samples. In general, a higher antimicrobial sensitivity against gram positive bacteria was observed for antimicrobial compounds related to groups A to I. Moreover, the antimicrobial sensitivity against gram positive pathogens was observed for antimicrobial compounds related to a wider spectrum (N to Q; Table 6). Regarding the antimicrobial resistance patterns of the isolated pathogens, *S. aureus* was the most resistant against the antimicrobial compounds together with *Bacillus* spp. The less resistant pathogens against the antimicrobial compounds belonged to Coliforms (gram negative) and to CNS and *Streptococcus* spp. (gram positive) (Table 6).

Table 7 shows the different MPFF sensitivity patterns of the isolated pathogens (*S. aureus* and CNS) obtained in in vitro cultures derived from milk samples. In general, in both pathogen groups, the antimicrobial sensitivity increased as a dose-specific response. The higher the MPFF dose applied, the higher the antimicrobial sensitivity, especially for *Staphylococcus epidermidis*, *Staphylococcus saprophyticus*, *Staphylococcus cohnii*, and *Staphylococcus xylosus*. Intermediate sensitivity against the MPFF compounds was observed for *Staphylococcus haemolyticus*, *Staphylococcus chromogenes*, and *Staphylococcus simulans*. On the other hand, *S. aureus* remained more resistant, even at the highest MPFF dose (Table 7).

### 3.6. Effect of MPFF Intramammary Infusion on the Cure Rate (%) in Mastitis-Diagnosed Quarters Naturally-Infected by Staphylococcus spp. in the Late Lactation

As stated before, a total of 200 quarters diagnosed as mastitis-positive (100 infected by *S.aureus* and 100 by CNS) were analysed. Twenty-five quarters were involved for each treatment (total 75 quarters) and were treated using MPFF at different doses (low: 500 mg; medium: 1000 mg; high: 1500 mg; 25 each). Twenty-five quarters were used as positive controls (*S. aureus*-derived and CNS-derived mastitis; 25 each). Cases were considered resolved if the scoring parameters for mastitis assessment achieved 0, <200,000 cells/mL, and 0 CFU/mL for CMT, SCC, and TBC, respectively, on day 3 post-partum. Regarding the positive controls, the spontaneous cure rate (%) was 4.0% (1/25) and 12.0% (3/25) for quarters infected by *S. aureus* and CNS, respectively. The overall cure rate (%) including all treatments was 14.6% (11/75) and 40.0% (30/75) for *S. aureus*-derived and CNS-derived mastitis, respectively (Table 8). Low, medium, and high MPFF dose treatments were successful in 4.0% (1/25), 12.0% (3/25), and 28.0% (7/25) of infected quarters by *S. aureus*, respectively. Regarding the quarters infected by CNS, the overall cure rate was 16.0% (4/25), 36.0% (9/25), and 68.0% (17/25) for the low, medium, and high MPFF dose, respectively. Differences among the cure rates in low, medium, and high MPFF-dose treatments were observed, irrespective of the pathogen agent (*p* < 0.05; Table 8).

## 4. Discussion

The occurrence of mastitis in livestock is associated with important economic traits such as reduced milk sales, increased use of antimicrobials, and elevated risk of culling [2,3]. The present research showed for the first time the effects of the intramammary infusion of MPFF in mastitis-diagnosed dairy cows to elucidate the role of MPFF as an alternative therapeutic compound for quarters naturally infected by *Staphylococcus* spp. in the late lactation. The assessment of CMT, SCCs, and TBCs values over the treatment course is decisive for the therapy success when using MPFF in mastitis-positive quarters. However, our results demonstrate that mastitis therapy success is strongly influenced by the pathogen species affecting dairy cows in the late lactation stage. Mastitis prevention and treatment in the late lactation period may play a critical role in future udder health and production [40,42,43,57]. However, unlike for other chemical-derived therapeutic products [9,58], there is scarce information related to the use of natural therapeutic compounds to treat and prevent mastitis in dairy cattle [59]. Alternative substances such as diosmin could be an interesting choice to treat mild mastitis cases because of its antimicrobial, phlebotonic, vascular-protecting, and anti-inflammatory activities [51]. In the present study the MPFF-derived effects regarding CMT, SCCs, and TBCs values improved as the concentration of the compound increased. Interestingly, these results are consistent with previous studies carried out by our research group when similar effects in metritis-positive dairy cows naturally infected by *E. coli* in the early post-partum period were observed using intrauterine MPFF infusions [60].

Regarding the negative control groups (healthy quarters), the CMT, SCCs, and TBCs values remained stable during the therapeutic assays while in mastitis-positive controls (infected quarters) and low MPFF-dose groups, the CMT, SCC, and TBC values gradually increased after drying-off and post-calving. Therefore, a remarkable dose-response effect was observed regarding CMT, SCCs, and TBCs being the lower MPFF-dose groups most affected by the lack of MPFF-derived effects. For that reason, at least medium and high MPFF doses were required to activate the antimicrobial and other protecting mechanisms in mastitis-diagnosed cows, irrespective of the pathogen producing the intramammary infection. Similar effects have been observed before in other studies when using chemical-derived products in mastitis therapy treatments showing important dose-response effects [61]. However, the causative agent could determine different dose-response effects [9]. In the present study, the MPFF treatment was more susceptible to failure in cows affected by *S. aureus*-derived infections, irrespective of the dose used. However, at higher MPFF doses, the therapeutic response based on CMT, SCCs, and TBCs values was far better in cows affected by CNS-derived infections. Similarly, higher MPFF doses were required in metritis-positive dairy cows to observe better therapeutic responses [60]. Thus, while CNS infected cows showed positive therapeutic effects after the use of a high MPFF dose (1500 mg), in quarters affected by *S. aureus* infection, it basically had no significant effect. In these quarters, the SCC was the only parameter which was slightly lower than before MPFF infusion. In general, *S. aureus* is more commonly associated with contagious mastitis than the other CNS species which are more associated with environmental mastitis [62]. Although MPFF shows high levels of biosafety as other flavonoids [49,50], the authors are unaware if doses higher than 1500 mg would be more effective for resolving *S. aureus*-derived infections. Consequently, as shown in the present research, more assays based on MPFF dosing protocols will be needed to improve its efficacy for mastitis-diagnosed dairy cows naturally infected by *Staphylococcus* spp. in the late lactation, especially those produced by the most resistant pathogens.

The cure rate (%) differs among treatments and it depends on the MPFF dose used and on the pathogen that caused the infection. The negative controls consisting of mastitis-free cows remained unchanged with regard to the healthy status throughout assays. In general, glands infected with *S. aureus,* even treated at a greater MPFF dose, had lower bacteriological cure rates, remaining < 30% in *S. aureus*-derived infections and <70% in CNS-derived cases. However, obvious differences between the positive controls and MPFF-treated quarters were observed, irrespective of the causative agent. Thus, there is evidence supporting that a higher MPFF dose involved a higher udder recovery success, probably due to lower doses not being enough to solve the infection. Similarly, therapeutic results derived from other antimicrobials have been observed to be different depending on the chemical origin, type of pathology, dose, and species treated [4,9,63,64].

Even though mammary gland bacteriological profiles were pretty similar in mastitis-diagnosed cows during pre-treatment period, SCCs and TBCs values differed significantly. All quarters had one milk sample (duplicate) taken before MPFF treatments on day 0 (drying-off), and a second one (duplicate as well) at MPFF post-treatment on day 3 post-partum. In that specific time window, SCCs were elevated only in mastitis-diagnosed quarters, specifically in positive controls (*S. aureus* and CNS groups) and low MPFF-dose treated quarters while TBCs profiles (CFU/mL) were unaltered. On the other hand, mastitis-diagnosed quarters had SCCs values above those of negative control quarters during all experiments. In the present study, no effect of low and medium MPFF dose on the post-treatment SCCs value was observed. Post-treatment SCCs value was lower in quarters that successfully underwent a bacteriological cure. As the cure rate did not differ between low and medium MPFF treatments, it is not surprising that the post-treatment SCCs values did not differ.

Overall the effect of MPFF as a bacteriostatic/bactericide compound against different Staphylococci was not conclusive, maybe due to self-specific sensitivity to the compound, regardless of the dose or perhaps due to Staphylococci resistant strains, especially those identified as *S. aureus* [65]. According to normal TBCs values and species found in control samples, the bacterial ratio in term of coagulase-positive vs. coagulase-negative Staphylococci were unchanged through the assay.

In the present research, high MPFF concentrations have been applied as an efficient dose to control SCCs as well as TBCs in cattle, as is similar to other chemical compounds [58,59,66]. The cure rates after MPFF treatment at high doses observed in mastitis-diagnosed quarters could hypothetically be traced back to the MPFF dose and the early mastitis diagnosis. Although MPFF was administered at a different intramammary dose, it might specifically improve the immunological response via cellular receptors related to the local defense due to the enhancement of the activity of immune cells when they leave the teat wall and enter the teat cistern to intercept bacteria protecting the mammary gland and increasing SCCs values [67,68]. This effect might not be related only to the local tissue defense, but the humoral immunological response related signs would be affected as well due to the endogenous MPFF-derived effects. Moreover, the cellular immunological response would require crossing the mammary gland epithelium barrier inducing the desired effects to protect against infections and increasing the rhythm of the bacteriological cure [51,69]. As there is no evidence that MPFF can come across the mammary gland epithelium, the possibility remains that MPFF applied to infected quarters might have a local effect at a certain time window. Although important differences have been described among antimicrobial compounds as conventional treatments in quarters affected by subclinical mastitis [9,59,70], information about the effects of MPFF treatment in mastitis-affected dairy cows and its relation to the CMT score, SCCs profile, and TBCs changes, particularly in the late lactation period, is not available. The possibility that MPFF could cross the epithelial barrier at higher doses would explain the increased SCC-related effects on the mammary gland found in the present research via immune cell receptors (e.g., via cytokines and chemokines) together with transcriptional factor modifications related to the major histocompatibility complex (MHC) during the inflammation process (e.g., modifying the expression of inflammatory-related genes such as *CXCL17*, *CX3CL1*, *CXCL16*, *CCL28*, *IL34*, *IL10RB*, *HVEM*, *FN14*, *CSF1*, and *OPG*) [71,72,73,74]. Other important factor influencing the cure rates of mammary gland infection could be related to several flavonoid-derived functions, such as their role as potent antioxidants attenuating and repairing the tissue damage, as well as several beneficial properties related to different inflammatory diseases [75,76]. In mammal species, immune cell receptors trigger the arrival of lymphocytes at the site of infection together with the interleukin-derived immune response (IL-1 α, β, and IL-6) [77,78]. On the other hand, the reactive oxygen species (ROS) and different proteases increase the tissue damage and fibrosis contributing to the local inflammation-derived effects found in mastitis, causing a subsequent state of irreversible atrophy [79,80,81,82]. Therefore, more studies are needed by using different MPFF compound ratios and doses to unravel molecular details of these effects. Moreover, the inhibition of free radicals derived from flavonoids may include several mediators such as prostaglandins, thromboxanes, and leukotrienes, inhibiting several genes such as *PLA2*, *COX*, and *LOX* [83,84,85,86]. The inflammatory process mediated by flavonoids inhibits signaling transduction pathways and secretory processes [87] together with a decreased cell proliferation and a lower release of pro-inflammatory cytokines [48,88]. Moreover, the flavonoids inhibit the cell maturation by suppressing the response of CD4 + T cells and diminishing several IL-family effects through blocking IL-cytokine and IgE receptors [88,89]. Therefore, MPFF might also activate or inhibit other receptors located in different tissues such as the mammary gland; however, this has yet to be described.

In the present study, the MPFF effect in the mammary gland tissue seems to be efficient at medium-term, contrary to conventional antimicrobial effects due to obvious dose-specific effects which were observed. More assays are needed, applying different protocols and MPFF concentrations. In case of CNS-derived mastitis, healthy quarters from negative control group had preserved CMT, SCCs, and TBCs values similar to those values associated with the use of high MPFF dose. However, in the case of *S. aureus*-derived mastitis, all parameters were significantly different compared with those belonging to the negative control and pretty similar to those obtained in the positive control. Similar to mastitis-free quarters (negative control), within the mastitis-diagnosed quarters, the higher MPFF doses showed better results stimulating cure rates which may explain the effects of MPFF on CMT, SCCs, and TBCs obtained in CNS-derived cases. However, when negative control was compared to *S. aureus*-derived cases, higher MPFF doses showed no better results for all parameters, especially at lower and medium MPFF doses. It is believed that the lack of cure during the dry-off period is related to the intracellular accumulation of *S. aureus* within leukocytes; however, intermittent and cyclical shedding of *S. aureus* frequently occurs [90,91]. Therefore, the MPFF use was beneficial in *S. aureus*-derived cases but not fully effective. Thus, mastitis-diagnosed quarters had more difficulties obtaining better recovering rates when biofilm-producing microorganisms such as *S. aureus* were the causative agents, so it could be inferred that the effects of MPFF were determined by the initial udder health status of the quarters treated, as stated before for other antimicrobial compounds [92,93]. Nevertheless, treatment failure to remove pathogens may be associated not only with the pathogen species, antimicrobial used, or initial udder health status, but even with the administration route, antimicrobial resistance, therapy duration, mastitis severity grade, and lack of immune response [4,36,94,95].

Based on the obtained findings, MPFF may be a natural alternative for clinical mastitis control, increasing the cure rates and reducing the treatment-derived costs (MPFF is inexpensive compared to other compounds) in *Staphylococcus* spp. mastitis-positive cases in dairy cattle. MPFF could be used as a new choice against antimicrobial resistance prevalence which threatens animal and human welfare and health, food safety, environment, and economic development [36]. In addition to MPFF, other specific natural compounds obtained recently from plants showed similar clinical implications for mastitis treatment and prevention and should also be taken into account for future applications in Staphylococci-derived infections in dairy cattle [96,97].

## 5. Conclusions

In conclusion, MPFF bactericidal and protective effects seem to be more efficient when CNS-derived mastitis was treated at higher doses. However, regarding *S. aureus*-derived mastitis, the MPFF dose would need to be adjusted in order to improve the cure rates. The antibiogram-derived results support the fact that dose-specific protocols during the MPFF intramammary administration should be based on considering the particular sensitivity observed in the SCCs and TBCs responses. Finally, the use of MPFF may be useful as a moderate bacteriostatic and bactericidal compound and should be differentially applied depending on the mastitis causative agent, since coagulase-negative Staphylococci were more sensitive to MPFF antimicrobial effects than coagulase-positive *S. aureus*; however, further research is needed on the effects of MPFF application in mastitis-positive dairy cows.

## Figures and Tables

**Table 1:** Identification of all experimental groups in the present study.

Experimental Groups									
Control Groups				Treatment Groups					
**Negative Control (Healthy Non-Treated;** ***n* = 50)**		**Positive Control (Mastitis Non- Treated; *n* = 50)**		**Low Dose (Mastitis-Treated MPFF: 500 mg; *n* = 50)**		**Medium Dose (Mastitis-Treated MPFF: 1000 mg; *n* = 50)**		**High Dose (Mastitis-Treated MPFF: 1500 mg; *n* = 50)**	
**-** **(*n* = 25)**	**-** **(*n* = 25)**	***Staph. aureus*** **(*n* = 25)**	**CNS** **(*n* = 25)**	***Staph. aureus*** **(*n* = 25)**	**CNS** **(*n* = 25)**	***Staph. aureus*** **(*n* = 25)**	**CNS** **(*n* = 25)**	***Staph. aureus*** **(*n* = 25)**	**CNS** **(*n* = 25)**

**Table 2:** Identification of microorganisms from in vitro cultures in milk samples and potential pathogenicity in producing mastitis in the late lactation.

Bacterial Species/Type	Associated to Frequent Mastitis/Considered Pathogen		Prevalence (% Total)	Prevalence (% Mastitis+)
**GRAM−**				
***Enterobacter* spp.**	No	Yes	4/250 (1.6%)	4/200 (2.0%)
Coliforms (≠spp.) *	Yes	Yes	39/250 (15.6%)	39/200(19.5%)
***Citrobacter freundii***	No	Yes	3/250 (1.2%)	3/200 (1.5%)
*Escherichia coli* *	Yes	Yes	51/250 (20.4%)	51/200 (25.5%)
***Klebsiella* spp.**	No	Yes	7/250 (2.8%)	7/200 (3.5%)
***Pseudomonas aeuroginosa***	No	Yes	5/250 (2.0%)	5/200 (2.5%)
***Proteus mirabilis***	No	Yes	2/250 (0.8%)	2/200 (1.0%)
***Yersinia* spp.**	No	Yes	6/250 (2.4%)	6/200 (3.0%)
***Salmonella* spp.**	No	Yes	3/250 (1.2%)	3/200 (1.5%)
***GRAM+***				
*Staphylococcus aureus* *	Yes	Yes	63/250(25.2%)	63/200 (31.5%)
CNS *	Yes	Yes	46/250 (18.4%)	46/200 (23.0%)
*Staphylococcus chromogenes* *	Yes	Yes	26/250(10.4%)	26/200 (13.0%)
***Staphylococcus xilosus***	No	Yes	18/250(7.2%)	18/200 (9.0%)
***Staphylococcus simulans***	No	Yes	15/250(6.0%)	15/200 (7.5%)
***Staphylococcus cohnii***	No	Yes	2/250(0.8%)	2/200 (1.0%)
***Staphylococcus epidermidis***	No	Yes	16/250 (6.4%)	16/200 (8.0%)
*Staphylococcus haemolyticus* *	Yes	Yes	20/250(8.0%)	20/200 (10.0%)
***Staphylococcus saprophyticus***	No	Yes	3/250 (1.2%)	3/200 (1.5%)
***Micrococcus* spp.**	No	Yes	2/250 (0.8%)	2/200 (1.0%)
*Bacillus* spp. *	Yes	Yes	14/250 (5.6%)	14/200 (7.0%)
***Enterococcus* spp.**	No	Yes	5/250(2.0%)	5/200 (2.5%)
***Corynebacterium* spp.**	No	Yes	6/250 (2.4%)	6/200 (3.0%)
***Listeria monocytogenes***	No	Yes	8/250(3.2%)	8/200 (4.0%)
*Streptococcus agalactiae* *	Yes	Yes	38/250(15.2%)	38/200 (19.0%)
Streptococcus dysgalactiae *	Yes	Yes	32/250(12.8%)	32/200 (16.0%)
*Streptococcus uberis* *	Yes	Yes	18/250 (7.2%)	18/200 (9.0%)
**Total**	357/1227 (29.1%)		357	357

Relative prevalence (%) calculated taking into account all experimental quarters (No. pathogen cases divided by positive + negative quarters, total 250). Relative prevalence (%) within mastitis cases calculated considering only experimental positive quarters (No. pathogen cases divided by positive quarters, total 200). CNS *: only considered *S. chromogenes* + *S. haemolyticus.* Asterisk (*): frequent + considered pathogen.

**Table 3:** MPFF infusion and the somatic cell counts (SCCs; × 10^6^ cells/mL) from milk samples on day 0 (pre-treatment) and on day 3 post-calving (post-treatment) in mastitis-diagnosed quarters naturally infected by *Staphylocuccus* spp. in the late lactation.

Somatic Cell Counts (SCCs: 10^6^ × cells/mL)										
Timepoints	(−) Control (*n* = 25; Healthy Non-Treated)		(+) Control (*n* = 25; Mastitis Non-Treated)		Low Dose (*n* = 25; Mastitis MPFF: 500 mg)		Medium Dose (*n* = 25; Mastitis MPFF: 1000 mg)		High Dose (*n* = 25; Mastitis MPFF: 1500 mg)	
	*Staph. aureus*	CNS	*Staph. aureus*	CNS	*Staph. aureus*	CNS	*Staph. aureus*	CNS	*Staph. aureus*	CNS
**Day 0 Drying-Off** **(Pre-Treatment)**	0.10 ^Aa^	0.09 ^Aa^	0.34 ^Ba^	0.40 ^Ba^	0.43 ^Ba^	0.38 ^Ba^	0.44 ^Ba^	0.39 ^Ba^	0.36 ^Ba^	0.46 ^Ba^
**Day 3 Post-Calving** **(Post-Treatment)**	0.13 ^Aa^	0.11 ^Aa^	0.39 ^Ba^	0.38 ^Ba^	0.50 ^Ca^	0.41 ^BCa^	0.39 ^Ba^	0.28 ^DEb^	0.32 ^BDa^	0.21 ^Eb^
**Difference bt/at** **(10^6^ × cells/mL)**	+0.03	+0.02	+0.05	−0.02	+0.07	+0.03	−0.05	−0.11	−0.04	−0.25

SCCs (×10^6^ cells/mL) in milk samples collected on day 0 (pre-treatment) and on day 3 post-partum after MPFF treatment in mastitis-diagnosed quarters. Superscripts (A–E) within a row show statistical differences among groups (*p* < 0.05). Superscripts within a column (a,b) show differences among time points (*p* < 0.05). bt: before treatment; at: after treatment. CNS: coagulase-negative Staphylococci.

**Table 4:** MPFF infusion and the number of pathogen isolates on day 0 (pre-treatment) and on day 3 post-partum (post-treatment) in mastitis-diagnosed quarters naturally infected by *Staphylocuccus* spp. in the late lactation.

No. Pathogens-Isolates										
Timepoints	(−) Control		(+) Control		Low Dose		Medium Dose		High Dose	
	(*n* = 25; Healthy Non-Treated)		(*n* = 25; Mastitis Non-Treated)		(*n* = 25; Mastitis MPFF: 500 mg)		(*n* = 25; Mastitis MPFF: 1000 mg)		(*n* = 25; Mastitis MPFF:1500 mg)	
	*Staph. aureus*	CNS	*Staph. aureus*	CNS	*Staph. aureus*	CNS	*Staph. aureus*	CNS	*Staph. aureus*	CNS
**Day 0 Drying-Off** **(Pre-Treatment)**	0.0 ^Aa^ (0/25)	0.0 ^Aa^ (0/25)	2.5 ^Ba^ (63/25)	2.3 ^Ba^ (57/25)	2.8 ^Ba^ (71/25)	2.6 ^Ba^ (66/25)	2.4 ^Ba^ (60/25)	2.3 ^Ba^ (58/25)	2.3 ^Ba^ (59/25)	2.6 ^Ba^ (65/25)
**Day 3 Post-Calving** **(Post-Treatment)**	0.0 ^Aa^ (0/25)	0.0 ^Aa^ (0/25)	2.4 ^Ba^ (60/25)	1.8 ^Ca^ (45/25)	2.5 ^Ba^ (64/25)	1.9 ^Cb^ (47/25)	1.7 ^Cb^ (42/25)	1.0 ^DEb^ (25/25)	1.3 ^CDb^ (33/25)	0.7 ^Eb^ (17/25)
**Total Reduction** **(No. Isolates)**	-	-	3	12	7	19	18	33	26	48

Pathogen isolates on day 0 (pre-treatment) and on day 3 post-partum after MPFF treatment in mastitis-diagnosed quarters. Superscripts (A–E) within a row show statistical differences among groups (*p* < 0.05). Superscripts within a column (a,b) show differences among time points (*p* < 0.05). CNS: coagulase-negative Staphylococci.

**Table 5:** MPFF infusion and total bacterial counts (TBCs; ×10^5^ CFUs/mL) on day 0 (pre-treatment) and on day 3 post-calving (post-treatment) in mastitis-diagnosed quarters naturally-infected by *Staphylocuccus* spp. in the late lactation.

Total Bacterial Counts (TBCs: ×10^5^ CFUs/mL)										
Timepoints	(-) Control		(+) Control		Low Dose		Medium Dose		High Dose	
	(*n* = 25; Healthy Non-Treated)		(*n* = 25; Mastitis Non-Treated)		(*n* = 25; Mastitis MPFF: 500 mg)		(*n* = 25; Mastitis MPFF: 1000 mg)		(*n* = 25; Mastitis MPFF:1500 mg)	
	*Staph. aureus*	CNS	*Staph. aureus*	CNS	*Staph. aureus*	CNS	*Staph. aureus*	CNS	*Staph. aureus*	CNS
**Day 0 Drying-Off** **(Pre-Treatment)**	0.00 ^Aa^	0.00 ^Aa^	3.32 ^Ba^	2.89 ^Ba^	3.80 ^Ba^	3.48 ^Ba^	3.63 ^Ba^	3.87 ^Ba^	3.71 ^Ba^	3.69 ^Ba^
**Day 3 Post-Calving** **(Post-Treatment)**	0.00 ^Aa^	0.00 ^Aa^	3.60 ^BCa^	3.12 ^Ba^	4.22 ^Ca^	3.04 ^Ba^	3.34 ^Ba^	2.10 ^Db^	3.16 ^Ba^	1.72 ^Db^
**Difference at/bt** **(10^5^ × cells/mL)**	0	0	0.28	–0.23	0.42	–0.44	-0.29	–1.67	–0.55	–1.97

TBCs (×10^5^ CFUs/mL) in milk samples collected on day 0 (pre-treatment) and on day 3 post-partum after MPFF treatment in mastitis-diagnosed quarters. Superscripts (A–D) within a row show statistical differences among groups (*p* < 0.05). Superscripts within a column (a,b) show differences among time points (*p* < 0.05). CNS: coagulase-negative Staphylococci.

**Table 6:** Antimicrobial sensitivity patterns of representative pathogen species obtained from milk samples in mastitis-diagnosed quarters in the late lactation.

Bacterial spp./Types	Antimicrobial Sensitivity Patterns																			
	A	B	C	D	E	F	G	H	I	J	K	L	M	N	O	P	Q	R	S	T
***GRAM−***																				
**Coliforms (≠ spp.)**	++	++	++	++	++	+		−			++									
***Klebsiella* spp.**	++	++		++		−			−		++									−
E. coli *		++		+	−	−		++	+										+	
***GRAM+***																				
**Bacillus (≠ spp.)**	++	−		+	++	−		+	−		−			+	++		++			
**CNS**	++			++	++	++		+						−		+				
***Staph. aureus***	++	−		++	++	−	++	+	−		−			−	++		++	−		
***Strep. agalactiae***	++				+	++		−	++							++				−
***Str. dysgalactiae***	++				++	++		++	+							++				−
***Strep. uberis***	++				++	++		++	+							++				−
**Total HS (++)**	8	3	1	4	6	4	1	3	1	0	2	0	0	0	2	3	2	0	0	0
**Total R (−)**	0	2	0	0	1	4	0	2	3	0	2	0	0	2	0	0	0	1	0	4

Antimicrobial sensitivity patterns. (A) Cephalosporins A (include Cefuroxime, Cefoxitin, Ceftriaxone, Cefotaxime/Cefamandole, Cefmetzole, Ceftizoxime, Cefepime, Cefixime, Ceftazidime, Cefoperazone, Ceftibuten, Cefotetan, Ceftiofur); (B) Quinolones (include Norfloxacin, Ofloxacin, Enrofloxacin, Levofloxacin, Nalidixic acid, Gemifloxacin); (C) Carbapenems (include Imipenem, Meropenem); (D) Aminoglycosides (Netilmicin, Neomycin, Gentamicin, Amikacin, Sisomycin); (E) Cephalosporins B (include Cefalexin, Cefazolin, Cephalothin, Cefaclor, Cefadroxil, Cephradine); (F) Penicillins + β-lactamase inhibitors (Amoxacillin + Clavulanic acid, Ampicillin + Sulbactam); (G) Cephalosporins C (Cefotiam); (H) Sulfonamides + Others (Sulfonamides + Trimethoprim); (I) Penicillins I (Amoxicillin); (J) Glycopeptides (Teicoplamine); (K) Quinolones (Norfloxacin, Orfloxacin, Enrofloxacin, Moxifloxacin, Gatifloxacin); (L) Glycopeptides II (Vancomycin); (M) Others (Chloramphenicol, florfenicol); (N) Tetracyclins (Doxycyclicline, Oxytetracyclin, Tetracycline): (O) Macrolids (Erythromycin, Spiramycin); (P) Others II (Lincomycin, Clindamycin, Fusidic acid, Polymyxin B); (Q) Aminoglycosides II (Streptomycin); (R) Oxazolidinones (Linezolid); (S) Quinolones II (Ofloxacin, Levofloxacin, Gemifloxacin); T) Penicillins II (Ampicilin). Nomenclature: (−) resistant; (+) low/moderate sensitivity; (++) moderate/high sensitivity.

**Table 7:** MPFF sensitivity patterns of the pathogen species obtained from milk samples in mastitis-diagnosed quarters naturally infected by *Staphylocuccus* spp. in the late lactation.

Pathogen Species		Antimicrobial Sensitivity		
	(+) Control (*n* = 50; Mastitis Non-Treated)	Low Dose (*n* = 50; Mastitis MPFF: 500 mg)	Medium Dose (*n* = 50; Mastitis MPFF: 1000 mg)	High Dose (*n* = 50; Mastitis MPFF:1500 mg)
**Coagulase-negative *Staph*. (CNS)**				
***Staphylococcus haemolyticus***	−	−	+	++
***Staphylococcus epidermidis***	−	+	++	+++
***Staphylococcus saprophyticus***	−	+	++	+++
***Staphylococcus cohnii***	−	+	++	+++
***Staphylococcus chromogenes***	−	−	+	++
***Staphylococcus simulans***	−	−	+	++
***Staphylococcus xylosus***	−	+	++	+++
**Coagulase-positive *Staph.***				
***Staphylococcus aureus***	−	−	−	+

MPFF sensitivity patterns. Nomenclature: (−) resistant; (+) low sensitivity; (++) moderate sensitivity; (+++) high sensitivity.

**Table 8:** Effects of intramammary MPFF infusion on the cure rates (%) on day 3 post-calving (post-treatment) in mastitis-diagnosed quarters naturally infected by *Staphylococcus* spp. in the late lactation.

Experimental Groups	(-) Control (*n* = 25; Healthy Non-Treated)		(+) Control (*n* = 25; Mastitis Non-Treated)		Low Dose (*n* = 25; Mastitis MPFF:500 mg)		Medium Dose (*n* = 25; Mastitis MPFF:1000 mg)		High Dose (*n* = 25; Mastitis MPFF:1500 mg)	
	*Staph. aureus*	CNS	*Staph. aureus*	CNS	*Staph. aureus*	CNS	*Staph. aureus*	CNS	Staph. aureus	CNS
**Cure Rate (%) on Day 3 Post-Calving** **(Post-Treatment)**	0.0 ^A^ (0/25)	0.0 ^A^ (0/25)	4.0 ^A^ (1/25)	12.0 ^AB^ (3/25)	4.0 ^A^ (1/25)	16.0 ^B^ (4/25)	12.0 ^AB^ (3/25)	36.0 ^C^ (9/25)	28.0 ^C^ (7/25)	68.0 ^D^ (17/25)
*Staph. aureus*							CNS			
**Total Cure Rate (%) including All Treatments**	11/75 ^A^ (14.6%)						30/75 ^B^ (40.0%)			

Cure rate (%) on day 3 post-partum after MPFF treatment in mastitis-diagnosed quarters. Superscripts (A–D) within a row show statistical differences among groups (*p* < 0.05). CNS: coagulase-negative Staphylococci.

## Data Availability

Not applicable.
